# A Psychometric Validation of the Korean Version of Disaster Response Self-Efficacy Scale for Nursing Students

**DOI:** 10.3390/ijerph20042804

**Published:** 2023-02-04

**Authors:** Sung Hae Kim

**Affiliations:** Department of Nursing, College of Health, Welfare and Education, Tongmyong University, Busan 48520, Republic of Korea; sunghae@tu.ac.kr; Tel.: +82-51-629-2673

**Keywords:** disasters, self-efficacy, nursing students, psychometrics, Rasch analysis

## Abstract

Nurses are very important healthcare providers in disaster situations, and from undergraduate nursing students to professional registered nurses, such practitioners should focus on strengthening their disaster-response-related self-efficacy and competency. The purpose of this study was to develop a Korean version of the Disaster Response Self-Efficacy Scale (DRSES-K) and evaluate its psychometric properties. The DRSES was translated into Korean and developed based on the translation and adaptation of instruments suggested by the World Health Organization. Data were collected from 30 October to 23 November 2020. A total of 209 undergraduate nursing students participated in this study. Psychometric properties were assessed using the programs SPSS/WIN 29.0, AMOS 26.0, and Winsteps 3.68.2, with which Rasch model analysis was carried out. The DRSES-K fit was sufficiently suitable for the unidimensional Rasch model with acceptable goodness of fit (χ^2^/df = 2.20 (*p* < 0.001), CFI = 0.92, IFI = 0.92, TLI = 0.91, AGFI = 0.82, and RMSEA = 0.07). The DRSES-K was significantly correlated with the measure of preparedness for disaster response, so concurrent validity was satisfied. The findings in this study suggested that the DRSES-K is a scale with verified validity and reliability. It is expected that the DRSES-K will be used for disaster nursing education to strengthen the competency of undergraduate nursing students.

## 1. Introduction

In the past, the frequently occurring disasters have mainly been natural disasters such as typhoons, floods, earthquakes, and heavy snow. However, with the emergence of Middle East Respiratory Syndrome in 2015 and Corona Virus Disease-19 (COVID-19) in 2019, we now face serious social disasters caused by infectious diseases [1,2]. Across the globe, the incidence of various disaster-related accidents is increasing every year, with such disasters threatening to cause physical, psychological, and social damage [1,3]. Systematic disaster management at the national level must be established, and a rapid response to disaster situations should be activated in such circumstances. Healthcare providers with disaster response capabilities play an essential role by providing rapid emergency medical services such as emergency triage, transfer, and treatment of patients in disaster situations [1,3,3].

The COVID-19 pandemic has highlighted the importance of nursing staff and emphasized their roles and responsibilities. Therefore, from undergraduate nursing students to registered nurses in clinics and the community, training and education are required to strengthen nursing competency with respect to responding to disasters [4,5]. Since 2008, the World Health Organization (WHO) has emphasized the importance of disaster nursing competence and stated that disaster nursing education is essential for undergraduate nursing students [6,7,8]. The International Council of Nurses (ICN) announced the ‘Framework of Disaster Nursing Competencies’ and emphasized the integrated implementation of disaster-related nursing competencies [7,8,9]. Therefore, there is a need to develop a valid instrument with which to measure educational outcome and establish a disaster nursing education system to enhance nurses’ disaster nursing competency.

In previous studies on undergraduate nursing students, disaster preparedness [10,11,12], general self-efficacy [11,13] or disaster response self-efficacy [12,13], and disaster-related attitude [14] and knowledge [10] were significant influencing factors in terms of improving disaster nursing competency. Disaster preparedness and disaster response self-efficacy are the most important factors and are frequently used measurement variables when evaluating the effectiveness of disaster nursing programs [11,12,13,14,15]. In particular, disaster response self-efficacy affects an individual’s ability to engage in a disaster situation and respond appropriately when a disaster occurs [16]. Nursing students who participated in a disaster nursing education program significantly improved their post-education disaster response self-efficacy [12,13]. Therefore, it was found that nursing students with greater disaster response self-efficacy had higher disaster preparedness and competency [15,17]. Li et al. [18] emphasized the need for measurement tools with which to evaluate educational outcome while simultaneously developing and expanding disaster nursing training or programs for nursing students. The ‘Preparedness for Disaster Response’ procedure developed by Schmidt et al. [19] is widely used [10,11,12]. On the other hand, the lack of standardized tools for measuring the disaster response self-efficacy of nursing students has been noted [18]. Although general self-efficacy is used as an evaluation indicator, disaster response self-efficacy should reflect the specificity of disaster situations, which is different from general self-efficacy [12,18]. The Disaster Response Self-Efficiency Scale (DRSES] developed by Li et al. [18] is a measurement of the self-efficacy of disaster response designed for undergraduate nursing students. The DRSES has been translated into Arabic [16] and Turkish [20] and is being used as a scale for evaluating disaster nursing education among undergraduate nursing students.

Therefore, this study aims to develop and verify the psychometric validities of the Korean version of the Disaster Response Self-Efficiency Scale (DRSES-K). The DRSES-K can be used for educational purposes in order to strengthen disaster nursing competency for undergraduate nursing students in South Korea. In addition, the scale aims to provide basic data for the development of disaster response self-efficacy and disaster nursing educational programs for nursing college students by identifying and verifying the psychological and hierarchical properties of the employed measurement tool according to cultural intersection. Moreover, it is provided as evidence for further research on the development of instrument development by identifying and verifying the psychometric properties of the measurement tools according to cross-cultural contexts.

## 2. Materials and Methods

### 2.1. Research Design

This study incorporated a methodological design to test the psychometric properties of DRSES-K based on the processes of the translation and adaptation of instruments according to the guidelines proposed by the World Health Organization (WHO) [21].

### 2.2. Participants and Data Collection

The research participants were undergraduate nursing students. The inclusion criteria specified that the participants had to have been senior nursing students who understood the purpose of this study and voluntarily agreed to participate. The exclusion criteria were as follows: those who had limited communication abilities or could not use an online survey collection platform could not participate in the study. We recruited participants from three universities in South Korea using convenience sampling. The estimation of the minimum sample size with which to verify the validity of the instrument is stable when it is more than five times the number of questions, and at least 150 to 200 subjects should be secured to test construct validity [22,23]. The original DRSES developed by Li et al. consists of 19 items. Considering the maximum number of items in the DRSES-K, the required sample size was 229, including a dropout rate of 15%. A total of 229 subjects voluntarily participated using the online survey from 30 October to 23 November 2022. A total of 209 questionnaire data points were used for analysis, excluding 20 subjects for whom the Rasch model analysis was inappropriate [24,25].

### 2.3. Instruments

#### 2.3.1. General Characteristics

A questionnaire developed by a researcher based on previous research [10,11,16,18,20] related to disaster nursing was used. The questionnaire consisted of items concerning each subject’s age, gender, grade, disaster education experience, necessity of disaster nursing education, and willingness to participate in disaster nursing education.

#### 2.3.2. The Disaster Response Self-Efficacy Scale (DRSES)

DRSES is a scale developed by Li et al. [18] to measure the self-efficacy of disaster nursing students. The scale is composed of 19 items and 3 subscales, with the latter including ‘on-site rescue competency’, ‘disaster psychological nursing competency’, and ‘disaster role quality and adaptation competency’. The participants rate the items on a 5-point Likert scale ranging from 1 to 5, representing 1 = No confidence at all, 2 = Basically no confidence, 3 = Little confidence, 4 = Basically confident, and 5 = Complete confidence. A higher score indicates higher self-efficacy in responding to disasters. Li et al.’s [18] research reported an overall Cronbach’s alpha of 0.91. The reliability values of subscales were as follows: on-site rescue competency (Cronbach’s alpha = 0.89), disaster psychological nursing competency (Cronbach’s alpha = 0.86), and disaster role quality and adaptation competency (Cronbach’s alpha = 0.83).

#### 2.3.3. Korean Version of the Preparedness for Disaster Response

This study used Kim’s instrument [26], which is a Korean version of the disaster response readiness measurement tool developed by Schmidt et al. [19]. The scale consists of 15 items assessed through a 5-point Likert scale, wherein a higher score indicates a higher level of ‘Preparedness for Disaster Response’. Kim [26] reported that the Cronbach’s alpha they obtained was 0.87. In this study, the Cronbach’s alpha was 0.96, and McDonald’s omega coefficient was 0.93.

### 2.4. Scale Development Process

#### 2.4.1. Translation/Back-Translation Phase

This study carried out adaptation of the original DRSES into Korean based on the WHO’s translation and adaptation of instruments guideline. In the first stage, two nursing professors and one bilingual nursing professor independently translated the original scale from English into Korean. The three researchers compared the words, vocabulary, meaning, concepts, and clarity of the translation with the original tool and revised the scale according to the original’s cultural intersections with respect to Korean. In the second stage, back-translation was conducted by a bilingual Ph.D. student in nursing who has lived in the United States for more than 20 years and is fluent in both Korean and English. The Korean draft version of the scale was translated into English and then revised and confirmed by contrasting it with the original DRSES. Three experts with experience in disaster nursing and emergency medicine reviewed and confirmed the preliminary DRSES-K 19 questions through discussions, thereby completing this phase of the study.

#### 2.4.2. Content Validity Testing

The content validity of DRSES-K was verified using a Delphi survey, which consists of an expert panel with extensive knowledge and clinical experience in responding to disasters. A Delphi expert panel of 10 to 18 is appropriate [27], and 11 experts were selected in this study. Our panel comprised 4 professors of disaster and community nursing or adult health nursing, 2 registered nurses with master’s degrees who had more than 10 years of experience in disaster nursing education, 1 senior researcher of the Korea Institute of Radiological and Medical Sciences, 1 senior researcher of the Korea Institute of Disaster and Safety, 1 epidemiological investigator with a master’s degree in nursing, 1 professor of emergency medicine of Center for Disaster Relief Training and Research, and 1 senior researcher that was also a registered nurse of the Center for Disaster Rescue Training and Research. The data collection period spanned from 7 September to 11 October 2020. To rate the content validity, expertise was rated on 4-point Likert scale from 1 to 4, wherein each ranking was represented as follows: 1 = Not relevant at all, 2 = Not relevant, 3 = Relevant, and 4 = Very relevant. In this study, the content validity ratio (CVR) was selected as a suitable item according to the proposal of Ayre and Scally [28], and the cut-off index was CVR = 0.64 or higher. Two rounds of the Delphi survey were performed. In the first Delphi survey, the CVR value of ‘lifting’ was very low, namely 0.09. The meaning of ‘lifting’ is ambiguous, and the competence evaluated was the professional competence of paramedics in role of emergency rescue in a disaster situation rather than the competence of nursing students [29]. In the second Delphi survey, the CVR value of ‘Effective circumstances for post-disaster infectious diseases, acute addiction, etc.’ was low (0.46). This item was judged to correspond to the professional roles of APN with respect to infection control or those of epidemiological investigators, both exceeding the level of nursing students [30]. In addition, the Delphi panel gathered opinions among nursing students corresponding to the notion that the basic activities of identifying and preventing infectious diseases in disaster areas were more important than determining epidemiological circumstances in terms of disaster response. DRSES is a tool consisting of a total of 19 items separated into 3 subscales: on-site rescue competency (11 items), disaster psychological nursing competency (4 items), and disaster role quality and adaptation competency (4 items). According to the results of the two Delphi surveys, ‘lifting’ and ‘Effective circumstances for post-disaster infectious diseases, acute addiction, etc.’ were excluded. These two items were included in the ‘on-site rescue competency’ subscale of the original tool. The researchers and Delphi panel renamed the items in consideration of the characteristics of the items and subscales as follows: ‘disaster assessment and rescue competency’ (9 items), ‘disaster psychological nursing competency’ (4 items), and ‘disaster quality and adaption competency’ (4 items). Thus, the preliminary items of DRSES-K were completed. The CVR was 0.86, and a range of 0.64 to 1.0 was reported for each item.

#### 2.4.3. Preliminary Survey

A total of 10 fourth-grade nursing students participated in the pilot test to verify the participants’ understanding of the content and its clarity. The survey period was from 15th October to 17th October 2020. First, the preliminary DRSES-K questionnaire was completed. Short interviews were conducted concerning the participants’ levels of comprehension of the items and words regarding any difficulties in filling out the survey questionnaire. There were no items with ambiguous meanings, and the finalized DRSES-K comprised 17 items with 3 subscales.

### 2.5. Statistical Analysis

The collected data were analyzed using the SPSS/WIN 29.0 program, AMOS 26.0 program, and Winsteps 3.68.2 software. The general characteristics of participants were examined using descriptive statistics (i.e., mean and standard deviation). The construct validity; convergent, discriminant, and concurrent validity; and reliability of the DRSES-K were verified by conducting Rasch model analysis, confirmatory factor analysis (CFA), and internal consistency analysis.

#### 2.5.1. Rasch Model Analysis

Regarding construct validity, Rasch model analysis was conducted to determine the item fit, item difficulty parameter, item response category curves, separation index (SI), and reliability index (RI). The mean square (MNSQ) value was calculated and verified to determine the suitability of the model fit and evaluate whether an item satisfies the assumption of unidimensionality. If infit MNSQ value was satisfied from 0.5 to 2.0 and the point-measure correlation value was greater than 0.4, the model was interpreted to be appropriate at a suitable level [24,25]. Regarding the difficulty parameter of items, higher logit values of the measurement indicated more difficult items, and lower logit values indicated the easier items. The goodness of fit of the response category on the 5-point Likert scale was verified through item response category curves. SI ≥ 2.0 indicates that the unidimensionality of the item is suitable, and RI ≥ 0.80 is interpreted as satisfying the internal consistency of the scale [24,25].

#### 2.5.2. Confirmatory Factor Analysis

CFA was performed using the structural equation model. Model fit was analyzed according to the criteria in [23.24] If χ^2^/df ≤ 3.0, comparative fit index (CFI), Tucker–Lewis index (TLI), incremental fit index (IFI) ≥ 0.90, adjusted goodness-of-fit index (AGFI) ≥ 0.80, and root mean square error of approximation (RMSEA) ≤ 0.08 criterion were satisfied and factor loading was 0.40 or higher, the model was considered suitable [22,23]. 

#### 2.5.3. Convergent, Discriminant, and Concurrent Validity

The convergent and discriminant validity was tested by multitrait-multimethod matrix (MTMM). The MTMM is a method of analyzing the correlation between each item of a scale and subscale; it is used to verify the validity of components in scale development research [31,32]. A correlation coefficient between factors of 0.40 or higher indicates the satisfaction of convergent validity, and a 95% confidence interval of the correlation coefficient between factors that does not include 1.0 indicates the satisfaction of discriminant validity [31,32]. The concurrent validity was tested using Pearson’s correlation coefficients between the score of preparedness for disaster response and score of DRSES-K.

#### 2.5.4. Reliability

The reliability of the DRSES-K was analyzed by assessing internal consistency with the Cronbach’s alpha value and McDonald’s omega coefficient.

### 2.6. Ethical Considerations

This study collected data after obtaining approval from the Institute Review Board of the university (IRB No: 202005-HR-001). Using the online survey platform’s uniform resource locator (URL), the purpose, procedure, inclusion and exclusion criteria, anonymity, and confidentiality were explained on the initial page. Data were collected after obtaining informed consent from participants who voluntarily agreed to participate.

## 3. Results

### 3.1. General Characteristics of the Study Participants

The characteristics of the participants are shown in Table 1. A total of 209 fourth-grade nursing students participated in this study. The participants’ mean age was 23.19 ± 2.92 years, and most of the participants were female (87.1%). A total of 114 participants (54.5%) had experience in disaster-related education, 205 participants (98.1%) responded that they needed disaster nursing, and 201 people (96.2%) responded that they were willing to participate in a disaster nursing education program.

### 3.2. Construct Validity: Item Analysis Based on Rasch Model

**Figure 1:**
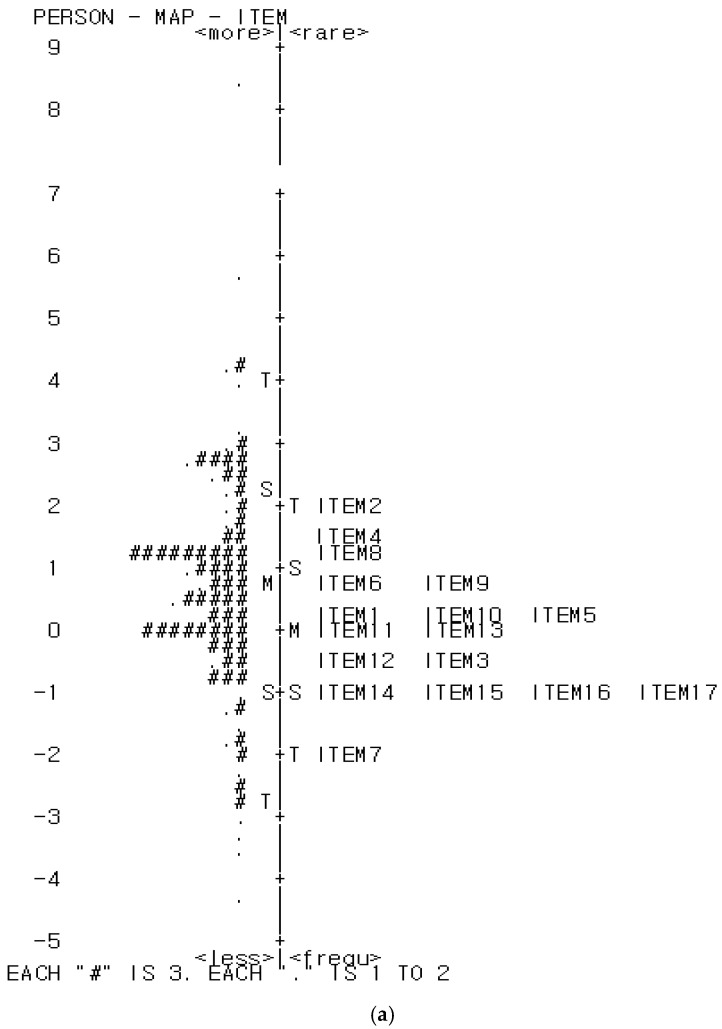

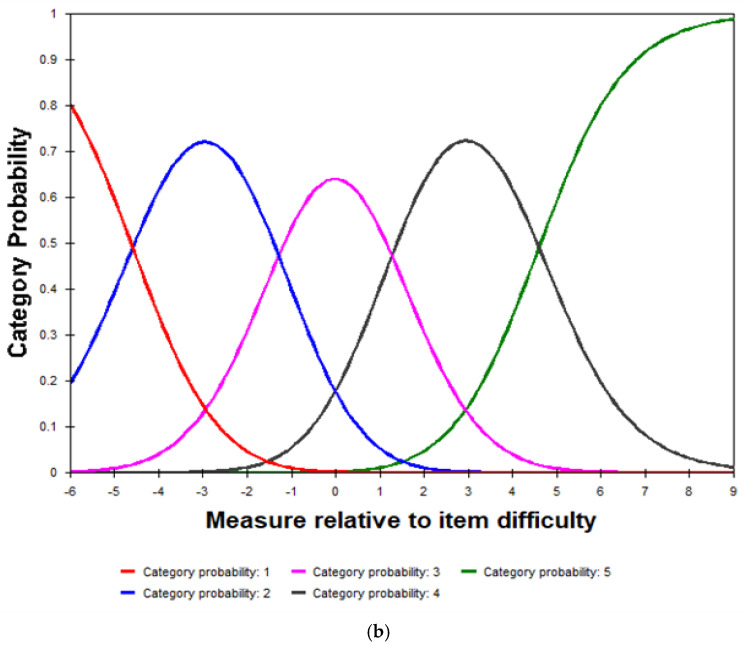
(**a**) Person item map for DRSES-K; (**b**) item response category curves of DRSES-K. M = Mean; S = 1 SD from the mean; T = 2 SD from the mean; SD = Standard deviation.

The results of the item analysis using the Rasch model are shown in Figure 1 and Table 2. The 209 data points collected in this study were used to analyze item fit via the Rasch model. The infit MNSQ ranged from 0.67 to 1.29, and the point-measure correlation ranged from 0.41 to 0.78, which satisfied the criteria for the goodness of fit of the item and showed that the 17 items of the DRSES-K were suitable for measuring the attributes of self-efficacy in disaster response. In order to verify the difficulty of the items, the measurement value was calculated. The measurement value ranged from −1.99 to 2.09. The ability level distribution and item level distribution of the participants were appropriately distributed within a similar category, thus confirming that the discrimination power of the DRSES-K was appropriate. Regarding the items’ difficulty, item 7 (Emergency rescue techniques, BLS), which had the lowest measured value, was the easiest item and corresponded to the highest self-efficacy of the participants. On the other hand, item 2 (Assess injuries accurately and swiftly), with the highest measured value, was the most difficult item and corresponded to the lowest self-efficacy of the participants (Figure 1a and Table 2). 

The item response category curves were tested to verify the suitability of the Likert rating scale. As a result of the analysis, the DRSES-K, composed of a 5-point Likert scale, reported that the item response category curves were appropriate. The rating scale was completely distinguished from each category, and the intersections between the scales appeared at relatively constant intervals (Figure 1b).

### 3.3. Construct Validity: Confirmatory Factor Analysis

**Figure 2:**
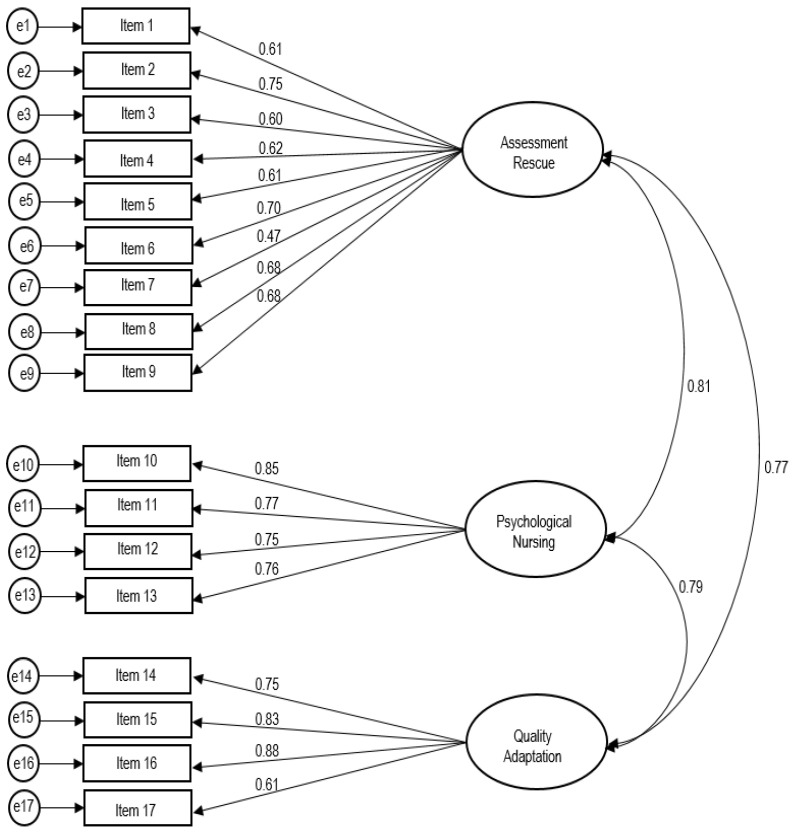
The confirmatory factor analysis of the DRSES-K model.

CFA was conducted to analyze construct validity. The commonality of the 17 items was from 0.41 to 0.82, which satisfied the criteria. The goodness-of-fit indices in the results of the analysis were found to be suitable. The model fit was achieved at an acceptable level, as follows: χ^2^/df = 2.20 (*p* < 0.001), Comparative Fit Index (CFI) = 0.92, Incremental Fit Index (IFI) = 0.92, Tucker-Lewis Index (TLI) = 0.91, Adjusted Goodness-of-Fit Index (AGFI) = 0.82, and Root Mean Square Error of Approximation (RMSEA) = 0.07 (Figure 2).

### 3.4. Convergent, Discriminant, and Concurrent Validity

MTMM was analyzed to verify the convergent and discriminant validity of the items of the DRSES-K (Table 3). The correlation coefficients of the 17 items ranged from 0.47 to 0.88, thereby demonstrating acceptability by achieving a level of 0.40 or greater, and the C.R value ranged from 6.20 to 12.49 (±1.97 or more, *p* < *0*.001), thus satisfying convergent validity. To verify the validity of these criteria, the correlation between the DRSES-K scores and the disaster response readiness scores was examined. The DRSES-K showed a significant positive correlation with disaster response readiness (r = 0.47, *p* < 0.001).

### 3.5. Reliability

In order to verify the internal consistency of the scale, the Cronbach’s alpha, McDonald’s omega coefficient, RI, and SI were tested. The overall Cronbach’s alpha and McDonald’s omega coefficient for the DRSES-K were the same at 0.93, which is considered an excellent level. The Subscales’ Cronbach’s alpha values were as follows: disaster assessment and rescue competency = 0.84, disaster psychological nursing competency = 0.86, and disaster quality and adaption competency = 0.85. Each item’s Cronbach’s alpha ranged from 0.92 to 0.93. In addition, the subscale’s McDonald’s omega coefficients were all the same at 0.86. As a result of the Rasch analysis, the SI values were 3.69 (participants) and 8.19 (items), and those of the RI were 0.93 (participants) and 0.99 (items). Therefore, the DRSES-K was identified as a highly reliable scale.

## 4. Discussion

Recently, we have experienced disaster situations such as the COVID-19 pandemic, typhoons, and earthquakes. Furthermore, it is impossible to predict the occurrence of all disasters. In a disaster situation, a rapid disaster response system is paramount, and efforts are required to deploy healthcare providers, especially nursing staff, and to strengthen their competency and disaster response self-efficacy. This methodological study was performed to verify the psychometric validity of the DRSES-K with respect to measuring South Korean undergraduate nursing students’ self-efficacy in term of disaster. The DRSES-K satisfied convergent, discriminant, and concurrent validity with high reliability.

In this study, the DRSES-K was developed according to the WHO’s translation and adaptation of instruments guideline [21]. As a result of the Delphi survey, two items were deleted, which were excluded for having low CVR values and low degrees of correlation with the attributes of self-efficacy in response to disasters. These items were ‘lifting’ and ‘Effective circumstances for post-disaster infectious diseases, acute addiction, etc.’. ‘Lifting’ was an inappropriate attribution because it was more focused on the role of paramedics or emergency rescue services in disaster situations rather than nursing students [29]. ‘Epidemiological assessment of post-disaster infectious diseases, acute poisoning, etc.’ is an important attribute in disaster nursing. However, epidemiological assessments, such as those applied to new infectious diseases or acute poisoning, require an integrated understanding of the geographical and social conditions of a given community [29], and such conditions were closely related to APN in infection control or epidemiological investigators [30]. It was observed that undergraduate nursing students in South Korea had a low level of preparedness for infectious disease in terms of disaster response [33]. Thus, greater educational efforts in this area are required. In order to improve disaster response competency and disaster response self-efficacy, we developed and provided education programs on the epidemiological assessment of post-disaster infectious diseases for undergraduate nursing students. The preliminary DRSES-K was composed of a different number of items from the original scale. The subscales were renamed in consideration of the items and the attributions’ subscales. In the previous studies that developed the DRSES adaptation scale [16,20], it can be seen that the subscales’ names were partially modified. When developing a translation scale, it is necessary to consider the cultural context, and the content validity of the scale can be increased through this process.

There are many nursing studies that have verified the validity of instruments based on the classical test theory (CTT) [34]. When the psychometric properties of the scale are tested based on the CTT, the item fit, item difficulty, and discrimination power are tested according to the characteristics of the subject at the time of the scale’s development. Therefore, it is necessary to consider that subscales and item attributes can be estimated differently depending on the other subjects [24,25]. Meanwhile ITT can estimate the parameters of an item attribute without being affected by the subject attribute [24,25,35]. Item response theory (ITT) has been used for the development and validation of instruments. As a result of Rasch model analysis, it was found that the infit MNSQ value of all the items in the DRSES-K satisfies the goodness-of-fit criterion. Therefore, the DRSES-K was suitable for measuring the attribute of self-efficacy in disaster response, and the item satisfied the assumption of unidimensionality [24,25].

As a result of analyzing the items’ difficulty, difficulty was ranked in the following order: item 2 (assess injuries accurately and swiftly), item 4 (triage technique), and item 8 (intensive care and nursing of critically ill patients). Items 2, 4, and 6 were difficult for nursing students to perform due to their high level of difficulty, which means that their self-efficacy in disaster response was low. Meanwhile, item 7 (emergency rescue techniques, BLS) was found to have the highest disaster response self-efficacy, as it was the easiest item to perform. This finding is similar to the results of previous studies [11,13,16], in which it was reported that undergraduate nursing students had difficulty assessing injured patients and identifying nursing problems even though they were aware of the roles of healthcare providers in disaster situations. However, ‘basic Life Support (BLS)’ is a required core nursing skill according to the Korean Accreditation Board of Nursing Education [36]. Undergraduate nursing students were found to have relatively high self-efficacy with respect to emergency rescue techniques compared to other items both because they were provided with sufficient education and training regarding cardiopulmonary resuscitation and due to their high skill proficiency. Disaster nursing education is important. As a result of this study, more than 98% of the participants responded that they needed disaster nursing education, and the willingness to participate in education was very high. This supports the findings of previous studies [11,26,37], wherein undergraduate nursing students had a high educational demand for triage technique and intensive acute care. In the United States, disaster nursing education was strengthened as the educational demand for disaster nursing increased following the 911 terrorist attacks [38]. In Japan, more than 60% of nursing colleges have already been providing disaster nursing education [39]. However, only 12.8% of nursing colleges provide ‘disaster nursing’ as a single subject within the nursing curriculum in South Korea [11,38]. In a previous study concerning undergraduate nursing students, the demand for simulation-based education was the highest as an educational method for disaster nursing [11,38,40]. Therefore, it is necessary to develop and spread simulation-based disaster nursing education programs, such as disaster and rescue activities, patient assessment and triage techniques in mass casualty incidents, and the acute care of emergency patients in disasters, to enhance disaster response competency and self-efficacy [11,38,39]. In addition, it will be possible to enhance competency and self-efficacy in disaster response through the multidisciplinary convergence of education and nursing.

As a result of confirmatory factor analysis, the model fit of the DRSES-K satisfied the goodness-of-fit index, and convergent, discriminant, and concurrent validity were also satisfied. The results supported the acceptability and validity of the structural factors and items of the developed DRSES-K. This finding is similar to previous studies that translated the DRSES-K into Turkish or Arabic [16,20]. Convergent, discriminant, and concurrent validity was not presented in the previous studies [16,20], including the original DRSES research conducted by Li et al. [18], so comparison with previous studies is limited. However, in this study, in which MTMM analysis was conducted along with Rasch model analysis, it was found that the DRSES-K satisfies the assumption of unidimensionality and convergent and discriminant validity. In addition, concurrent validity was verified by using the preparedness measure of the disaster response scale. This finding is consistent with the results of previous studies [10,11,12,22,23] in which it was found that nursing students’ disaster preparedness and disaster response self-efficacy are key to strengthening disaster nursing competency and can be improved through comprehensive disaster education and training. The developed DRSES-K is a highly reliable instrument that satisfies internal consistency. The level of Cronbach’s α in this study was similar to previous studies [16,18,20].

The limitations of this study are as follows. First, this study is limited in terms of the generalization of its research findings due to the use of convenience sampling. In further studies, we suggest evaluating the validity and reliability of the DRSES-K by various samples. Second, the DRSES-K verified reliability using Rasch model analysis and internal consistency analysis. However, in further studies, we suggest conducting a test-retest reliability examination. Third, although concurrent validity was presented in this study, further research with the goal of verifying the predictive validity of the DRSES-K is suggested using data such as disaster response competency, which should be evaluated by an independent evaluator and not through self-reporting, or patient outcomes in disasters. Fourth, this study was conducted with a cross-sectional design, and research results over time could not be presented. In the future, we recommend conducting longitudinally designed research on disaster response self-efficacy using the DRSES-K.

## 5. Conclusions

This study developed the DRSES-K to measure self-efficacy in disaster response and verified its psychometric properties among undergraduate nursing students in South Korea. This study provides meaningful findings, as item fit was tested using Rasch model analysis based on ITT. In addition, the development of the DRSES-K is expected to be useful for the discovery and consequent assessment of vulnerabilities by measuring how nursing students perceive disaster response self-efficacy in South Korea. As the importance of disaster nursing competency has been emphasized, it is necessary to establish a customized disaster nursing education system for college students in the nursing department. In further studies, we recommend using the DRSES-K to present effective educational measures to improve undergraduate nursing students’ self-efficacy in terms of responding to disasters.

## Figures and Tables

**Table 1:** General Characteristics of Participants (*N* = 209).

Characteristics	Categories	*N* (%)	
Age (years)		23.19 ± 2.92	
Gender	Male	27	(12.9)
	Female	182	(87.1)
Academic year	Senior	209	(100)
Experience in	Yes	114	(54.5)
disaster education	No	95	(45.5)
Necessity of	Yes	205	(98.1)
disaster nursing education	No	4	(1.9)
Willingness of	Yes	201	(96.2)
disaster nursing education	No	8	(3.8)

**Table 2:** Item Difficulty Measures and Fit Statistics applied in Rasch Model Analysis (*N* = 209).

Items	Measure	Infit MNSQ	Outfit MNSQ	PT-Measure CORR.
1. Detect the relative harm from the disaster	0.37	1.06	1.03	0.63
2. Assess injuries accurately and swiftly	2.09	0.85	0.84	0.72
3. Recognize vulnerable populations, such as chronic patients or disabled people	−0.39	1.06	1.08	0.62
4. Triage technique	1.58	1.11	1.10	0.59
5. Hemostasis, bandaging, and splinting	0.15	1.22	1.22	0.58
6. Transfer care	0.78	1.00	0.98	0.67
7. Emergency rescue techniques (BLS)	−1.99	1.29	1.33	0.41
8. Intensive care and nursing of critically ill patients	1.16	1.08	1.09	0.65
9. Prevention and control of infectious diseases in disaster area	0.78	0.99	0.99	0.66
10. Initial psychological assessment of disaster victims	0.13	0.79	0.78	0.75
11. Recognize common psychiatric and psychological problems after disaster, such as PTSD, depression, and anxiety	−0.06	1.07	1.04	0.65
12. Provide basic psychological intervention for disaster victims	−0.62	0.94	0.95	0.68
13. Referral of victims who need psychiatric and psychological treatment in the disaster area	0.08	0.87	0.88	0.75
14. Adjust one’s own psychological state and adapt to the working environment quickly	−1.01	0.97	0.98	0.69
15. Communicate with other team professionals and establish good cooperation relationship	−1.07	0.87	0.89	0.71
16. Actively communicate with victims and relatives and establish good nurse-patient relationship	−1.07	0.67	0.66	0.78
17. Obey professional ethics with humanitarian and full of empathy and love	−0.90	1.10	2.79	0.59

MNSQ = Mean Squared; PT-Measure CORR. = Point-Measure Correlation.

**Table 3:** Correlation Among Factors of DRSES-K (*N* = 209).

Model	r	*p*	95% CI	
			Lower CI	Upper CI
Assessment and rescue—Psychological nursing	0.695	< 0.001	0.681	0.759
Assessment and rescue—Quality adaptation	0.686	< 0.001	0.607	0.752
Psychological nursing—Quality adaptation	0.675	< 0.001	0.594	0.743

CI = Confidence Interval.

## Data Availability

Not applicable.
